# More than Entertainment: The Association of Social Media Exposure with Adolescents’ Preferences for and Consumption of Sugar-Sweetened Beverages

**DOI:** 10.3390/foods15122125

**Published:** 2026-06-12

**Authors:** Manjing Feng, Liuyang Yao

**Affiliations:** College of Economics & Management, Northwest A&F University, Yangling 712100, China; fengmanjing@nwafu.edu.cn

**Keywords:** social media, sugar-sweetened beverages, adolescents, preferences, consumption

## Abstract

Social media has become a significant factor in unhealthy consumption behaviors among adolescents, given the prevalent use of mobile phones and the internet. This study investigates the association between social media exposure and adolescents’ sugar-sweetened beverage (SSB) preferences, as well as their consumption behavior. This study included 1517 adolescents across Henan Province, China, in 2025. We employ a mixed logit model, a hurdle model, and an Ordinary Least Squares (OLS) model to assess the association of social media exposure with adolescents’ SSB preferences and consumption behavior. The findings indicate that social media exposure is positively associated with adolescents’ overall preference for SSB products. Specifically, it is associated with a higher preference for carbonated drinks and beverages containing sweeteners and a lower preference for juice. Furthermore, the association between social media exposure and SSB preferences differs between urban and rural adolescents. Rural adolescents exposed to social media tend to show a lower willingness to forgo SSB options, whereas urban adolescents exposed to social media tend to show less sensitivity to price attributes. Additionally, social media exposure is positively associated with both the selection and consumption of SSBs among adolescents, which in turn are linked to health concerns such as overweight and obesity.

## 1. Introduction

Adolescent health is critical to public health [1]. Unhealthy dietary behaviors can lead to an increased risk of cardiovascular diseases, diabetes, and other non-communicable diseases over time [2]. Although often overlooked in traditional nutritional interventions and considered a period of relative health, the adolescent developmental stage is receiving increasing attention as epidemiological evidence reveals unfavorable outcomes in areas such as weight gain, physical activity, and dietary intake [3]. Over the past four decades, the average BMI and obesity rates among children and adolescents have risen in most regions and countries, and weight gain during childhood and adolescence is highly likely to lead to lifelong overweight and obesity [4]. The growth rate of overweight and obesity in China is among the highest worldwide, with adults ranking 19th and children 12th globally [5]. Obesity in childhood tends to persist into adulthood, increasing the risk of type 2 diabetes, cardiovascular disease, and premature mortality [6]. These trends have been attributed to changes in the global food system, driven by dominant multinational food corporations. Foods and beverages that are highly processed, energy-dense, and palatable are not only marketed effectively but also aggressively promoted [7]. Based on sugar-sweetened beverage (SSB) intake data from the Global Dietary Database (2020), Chinese citizens consumed an average of 0.2 oz servings per week [8]. While this figure appears low on an individual basis, the aggregate consumption volume remains enormous given the size of the Chinese population. In 2019, the number of deaths attributed to sugar-sweetened beverages in China reached 46,633 with an increase of 95% to 1990 [9]. Given that SSBs are a significant source of added sugar in children’s diets [10], there is growing concern that regular consumption of free sugars increases energy intake, promotes a positive energy balance, reduces the intake of more nutritious foods, and leads to unhealthy diets and weight gain [11].

The marketing environment for food and beverages is considered a key driver behind the global rise in obesity rates [12]. Because cognitive development is not yet fully mature, children and adolescents are particularly susceptible to such food marketing [13]. Adolescence is a life stage with unique characteristics, making adolescents more vulnerable to food marketing than adults [14]. Marketing has been shown to be a significant driver of food and beverage choices among children and adolescents [15], leading them to prefer, purchase, and consume unhealthy foods and beverages [16]. The increase in childhood overweight and obesity has coincided with a rise in children’s consumption of soft drinks and snacks, both in frequency and average amount [17]. National surveys in China have found that the proportion of children aged 15–17 consuming sugar-sweetened beverages more than once per week increased sharply from 14.2% in 2002 to 79.2% in 2012 [18]; the consumption rates of SSBs (25%) among Chinese children and adolescents substantially exceed those of adults [19]. The nutritional quality assessments reveal that products promoted in social media marketing often contain excessive amounts of free sugars and saturated fats [20]. Therefore, this study explores the relationship between social media and adolescents’ beverage attribute preferences, as well as their consumption of sugar-sweetened beverages, which is of significant practical importance. Social media is not only an important channel for adolescents to access dietary information and consumption trends but also influences their perceptions of beverage taste, packaging, price, and health attributes. This study analyzes how social media exposure affects adolescents’ beverage attribute preferences, helping to reveal the underlying mechanisms behind sugar-sweetened beverage consumption behaviors. It also provides a theoretical basis for schools, families, and public health authorities to develop more targeted nutrition intervention strategies.

## 2. Literature Review

The evolution of digital marketing has introduced new challenges in capturing advertising exposure and its significant impact on dietary behaviors [21]. Digital marketing is highly targeted, interactive, often not recognized as advertising, and frequently accessed by children without supervision [22]. While television is the primary medium for food companies to advertise to children, evidence shows that companies are shifting their advertising expenditures to digital marketing and employing diverse promotional tactics to reach and engage young audiences, particularly on social networking platforms [23]. Adolescents now spend the majority of their non-television screen time on smartphones, with a large portion dedicated to social media platforms. A total of 95% of adolescents report having access to a smartphone, and 43% indicate they check social media hourly or “almost constantly” [24]. One study found that 82% of 12th graders reported daily social media use in 2016, compared to 52% in 2008 [25]. Marketing through social media can amplify the effects of television advertising on brand recall, liking, and reach at a significantly lower cost than traditional advertising [13]. Social media can be defined as the interaction or sharing of content among individuals via the internet or mobile platforms [26]. Adolescents are made particularly vulnerable to interactive promotions and advertisements for foods and beverages high in saturated fat, salt, or free sugars, due to the widespread popularity of social media and the development of digital marketing strategies [27]. Children and adolescents are exposed to unhealthy food and beverage marketing on social media applications from various sources, including advertisements, user-generated and celebrity-generated content, and other forms of entertainment content [23].

Adolescents face a risk of exposure to digital promotions for unhealthy foods and beverages, as food and beverage companies increasingly employ social media advertising and marketing strategies to engage younger audiences [28]. The advent of smartphones and tablets has significantly expanded the reach of food and beverage companies on digital platforms, which are not bound by the government and industry regulations that apply to traditional advertising [29]. Digital marketing may exert a stronger influence on consumer behavior than marketing in television and other traditional advertising environments for several reasons [30]. Children and adolescents represent an important consumer group for marketers, as they spend substantial amounts of their own money, significantly influence household spending, and represent future adult consumers [31]. Most product brands maintain their own social media pages, and sugar-sweetened beverage brands are among the most popular. Evidence shows that food advertising can stimulate respondents’ preferences and desires under laboratory conditions [32], and such engagement may increase the risk of consuming unhealthy foods and beverages. Although unhealthy food marketing is prevalent on social media and adolescents are highly engaged online with such brands [24], the understanding of how digital food marketing influences their dietary behaviors remains under development [33]. Additionally, many studies have focused on the impact of individual characteristics on adolescents’ SSB intake. It is possible that boys may present more heterogeneity in beverage consumption [34]. Parental educational attainment may influence household rules concerning unhealthy foods as well as their availability within the home environment [35]. Previous studies have demonstrated a significant association between household availability and SSB consumption [36]. Furthermore, evidence from China suggests that preferences for sweetened beverages have increased significantly among children residing in rural areas [37]. In addition, adolescents who receive larger amounts of pocket money are more likely to purchase sugar-sweetened beverages [38].

The existing literature has the following limitations. First, most studies only focus on the correlation between social media use and adolescents’ SSB consumption behavior, yet they fail to conduct a unified framework analysis of social media, beverage attribute preferences, and adolescents’ SSB consumption. Second, although discrete choice experiments (DCEs) have been used to measure adolescents’ preferences for beverage attributes, they are rarely combined with social media exposure. This study conducts two parallel analyses: the first one is an analysis of the association between social media exposure and SSB preferences; the second one is an analysis of the association between social media exposure and SSB consumption based on cross-sectional data. We explore the potential value of social media exposure in terms of both consumer preferences and choice. This integrated design helps us to understand the potential pathways linking social media to adolescents’ SSB consumption more comprehensively.

This paper contributes to the existing literature in three aspects. First, the findings suggest the potential value of making sugar content information a mandatory requirement in nutrition labeling systems. Second, we demonstrate that social media is significantly associated with adolescents’ preferences for SSB attributes. In particular, the heterogeneity of social media effects between urban and rural areas provides a theoretical basis for designing targeted measures based on the consumption preference characteristics of adolescents in different regions. Third, we verify the consistency of the correlation between social media use and adolescents’ SSB choice preferences and behaviors, providing insights for limiting social media use. The structure of this paper is as follows: In the subsequent section, we will introduce the experimental procedures used for the choice experiment. Then, we have an explanation of the data collection process, a description of the sample characteristics, a discussion of the empirical analysis of the data, and a presentation of the research findings and conclusions.

## 3. Research Design

This study investigates the association between social media exposure and adolescents’ sugar-sweetened beverage preferences and consumption. A discrete choice experiment was designed and administered alongside a questionnaire survey to collect relevant data. All analyses were performed using Stata 18. The following section details the survey procedures and data collection methods.

### 3.1. Data

This study employs a stratified random sampling method, drawing participants from two cities (Nanyang and Xuchang), four counties, eight schools, and thirty-two classes within Henan Province, China. The target population consists of adolescents in grades seven and eight. We distributed 1600 questionnaires. After removing outliers and incomplete responses, we obtained 1517 valid responses. A discrete choice experiment (DCE) was selected to elicit adolescents’ preferences of the attributes of sugar-sweetened beverages. This methodological choice is justified by two primary factors: first, the impending implementation of labeling regulations with mandatory sugar content in 2027 limits the availability of pertinent market data; second, the DCE framework effectively simulates decision-making in a grocery-shopping environment [39], thereby providing a robust approach for investigating consumer food preferences.

This study selects four attributes to describe the sugar-sweetened beverage products within the DCE drawing on the existing literature: price, sugar content, beverage type (tea drinks, carbonated drinks, juice, energy drinks), and sweetener type (no sweetener, natural caloric sweeteners, non-caloric artificial sweeteners). The specific levels for sugar content are established based on national and nutritional association classifications for low-sugar, sugary, and high-sugar beverages, corresponding to 4 g/100 mL, 9 g/100 mL, and 15 g/100 mL, respectively. The selection of beverage types focuses on four major categories with a substantial market presence. As for sweetener type, three categories are included: non-caloric artificial sweeteners commonly used in production, natural caloric sweeteners derived from sources like starch through complex processing, and no added sweetener.

**Figure 1:**
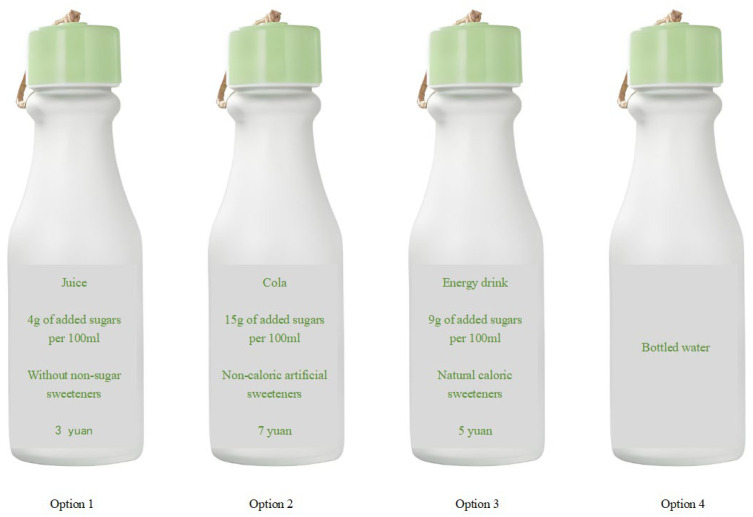
Sample choice situations.

A series of DCE tasks is designed to measure the influence of social media. Each participating student completes six choice scenarios to ensure orthogonality across the choice sets. As shown in Figure 1, every choice set presents three beverage alternatives (Option 1, Option 2, Option 3) and an opt-out alternative (Option 4). The opt-out option is defined as a non-sugar-sweetened beverage, specifically bottled water to enhance realism and reflect actual market conditions. Concurrently, we also collect data concerning the adolescents’ individual characteristics and household demographics. All data collection is conducted via in-person, paper-based questionnaire surveys.

### 3.2. Models

Choice modeling is typically derived from the assumption of utility-maximizing behavior by consumers, as formalized by Lancaster [40]. This theory posits that the utility of a good can be decomposed into separate utilities derived from its constituent characteristics or attributes. While researchers can observe some attributes of the alternatives and certain characteristics of individuals, a portion of individual utility remains unobservable and they are treated as stochastic. Since utility cannot be measured directly, indirect utility is inferred from observed preferences over product attributes. This study estimates consumer preferences for sugar-sweetened beverage products, drawing on random utility theory. Formally, the utility that respondent i derives from choosing alternative j in choice task t is given by

(1) $$U_{\mathrm{ijt}}=\beta_{i}^{\prime }X_{\mathrm{ijt}}+\varepsilon_{\mathrm{ijt}}$$

Here, $X_{\mathrm{ijt}}$ represents a vector of the observed attributes for alternative j, $\beta_{i}$ is the vector of utility weights, and $\varepsilon_{\mathrm{ijt}}$ denotes a random error term assumed to be independently and identically distributed (i.i.d.). Generally, the Conditional Logit (CL) model is widely employed as a baseline specification. This model treats parameters as fixed across observations and cannot account for heterogeneity in individual preferences. The Multinomial Logit (MNL) model is also commonly used to analyze discrete choice experiment data. However, the i.i.d. assumption leads to independence from the irrelevant alternatives (IIA) property. This imposes a limitation on the model’s practical application, as it fails to account for the degree of preference heterogeneity among participants [41]. It is more closely aligned with actual consumer behavior, as it allows for heterogeneity in preferences and provides greater flexibility in capturing substitution effects among products. Therefore, this study uses a mixed logit model to analyze adolescents’ preference for SSBs and the association between social media exposure and their choice preferences for SSBs. To estimate preferences for the four specified attributes—price, sugar content, beverage type, and sweetener type—the empirical model is specified as follows:

(2) $$U_{\mathrm{ijt}}=\beta_{p1}{\mathrm{price}}_{\mathrm{ijt}}+\beta_{a0}{\mathrm{out}}_{\mathrm{ijt}}+\beta_{a1}{\mathrm{sweetne}}_{\mathrm{ijt}}+\beta_{a2}{\mathrm{CocolBever}}_{\mathrm{ijt}}+\beta_{a3}{\mathrm{JuiceBever}}_{\mathrm{ijt}}\;\;+\beta_{a4}{\mathrm{EnergyBever}}_{\mathrm{ijt}}+\beta_{a5}{\mathrm{Nature}}_{\mathrm{ijt}}+\beta_{a6}{\mathrm{Artif}}_{\mathrm{ijt}}+\beta_{a7}{\mathrm{out}}_{\mathrm{ijt}}*{\mathrm{web}}_{i}\;\;\;\;\;\;\;\;\;\;\;\;+\beta_{a8}{\mathrm{price}}_{\mathrm{ijt}}*{\mathrm{web}}_{i}+\beta_{a9}{\mathrm{sweetne}}_{\mathrm{ijt}}*{\mathrm{web}}_{i}+\beta_{a10}{\mathrm{CocolBever}}_{\mathrm{ijt}}*{\mathrm{web}}_{i}\;\;+\beta_{a11}{\mathrm{JuiceBever}}_{\mathrm{ijt}}*{\mathrm{web}}_{i}+\beta_{a12}{\mathrm{EnergyBever}}_{\mathrm{ijt}}*{\mathrm{web}}_{i}\;+\beta_{a13}{\mathrm{Nature}}_{\mathrm{ijt}}*{\mathrm{web}}_{i}+\beta_{a14}{\mathrm{Artif}}_{\mathrm{ijt}}*{\mathrm{web}}_{i}+\varepsilon_{\mathrm{ijt}}$$

Here, β denotes the coefficient for each attribute chosen by each participant, i represents the participant, j denotes the choice task, and t indicates the choice scenario. ${\mathrm{out}}_{\mathrm{ijt}}$ is a specific constant term, indicating the utility when a consumer chooses “opt-out” among the three sugar-sweetened beverage options. When “opt-out” is selected, ${\mathrm{out}}_{\mathrm{ijt}}$ is assigned a value of 1; when any sugar-sweetened beverage product is chosen, ${\mathrm{out}}_{\mathrm{ijt}}$ is assigned a value of 0. ${\mathrm{price}}_{\mathrm{ijt}}$ is a continuous variable, referring to the three price levels included in the experimental design: 3 yuan, 5 yuan, and 7 yuan. ${\mathrm{sweetne}}_{\mathrm{ijt}}$ represents the sugar content of the sugar-sweetened beverage. Other dummy variables include beverage type (tea drinks, carbonated drinks, fruit juice drinks, energy drinks) and sweetener type (no sweetener, natural caloric sweeteners, non-caloric artificial sweeteners). $\varepsilon_{\mathrm{ijt}}$ is the stochastic error term, representing unobserved utility.

A hurdle model approach is employed as a secondary analysis to examine the association between social media exposure and adolescents’ selection of sugar-sweetened beverages. When respondents choose beverages across six choice tasks, the dependent variable is constructed by summing the number of times that a sugar-sweetened beverage is selected, resulting in a value ranging from 0 to 6. The hurdle model adopts a two-stage estimation process. The first stage models factors associated with selecting a sugar-sweetened beverage at least once, using a Probit model. The second stage models factors related to the non-zero frequency of sugar-sweetened beverage choices. The first stage models as the selection model, where the dependent variable is equal to one for adolescents who selected SSBs at least once and zero otherwise. For this second stage, a Poisson regression is estimated. This model incorporates social media exposure and respondent demographic data as independent variables to identify the characteristics associated with a higher frequency of selecting sugar-sweetened beverages.

To further investigate the association between social media exposure and adolescents’ SSB consumption, we specify the model as follows:

(3) $${\mathrm{beverage}}_{i}=\alpha_{0}+\alpha_{1}{\mathrm{web}}_{i}+\gamma_{1}X_{i}+\varepsilon_{i}$$

where ${\mathrm{beverage}}_{i}$ is the logarithm of adolescent’s SSB consumption, and this study performs a logarithmic transformation on SSB consumption. ${\mathrm{web}}_{i}$ denotes the daily duration of social media usage. $X_{i}$ consists of control variables, including individual-level characteristic variables and household-level characteristic variables.

## 4. Results

The final analytical sample comprises 1517 respondents. As shown in Table 1, the adolescents report average daily social media usage exceeding 3 h, and the sample contains an approximately equal proportion of males and females, all aged between 12 and 16 years. A total of 74.7% of the participants reside in rural areas, and 22.1% attend boarding schools. The average household size exceeds five members. Furthermore, the parents have an average age of 40 years and an average educational attainment above the junior high school level. The adolescents’ average weekly allowance is 32.817 Yuan. Their average Body Mass Index (BMI) is 19.22, which falls within the normal range. Finally, the availability of sugar-sweetened beverages at home is relatively low.

### 4.1. Social Media Exposure and Adolescents’ Preferences for SSBs

The choice experiment within this study contributes a total of 36,408 valid observations for analysis. This is derived from 1517 completed valid questionnaires, each containing six choice sets where respondents select from four alternative options. The results of the mixed logit model are presented in Table 2. In this model, all estimated coefficients are treated as random parameters following a normal distribution, except those for price and the opt-out alternative, which are specified as fixed.

First, the main effects are presented in column (1); both the price and opt-out coefficients are negative and statistically significant. It indicates that price is an important consideration for adolescent consumption, where higher prices correspond to lower preference. Furthermore, adolescents generally do not want to forgo the purchase of a sugar-sweetened beverage product altogether. The coefficient of sugar content is significantly negative, suggesting adolescents prefer beverages with lower sugar content. This finding demonstrates that sugar content information can contribute to achieving the goal of reducing adolescent SSB consumption. This attribute is included and analyzed via choice experiment due to market data limitations, as comprehensive regulations on sugar content labeling are not scheduled for full implementation until 2027. Adolescents exhibit a stronger preference for juice over tea drinks when considering different beverage types. In contrast, they exhibit a negative preference for both carbonated and energy drinks. Adolescents display a negative preference for both natural caloric and non-caloric artificial sweeteners when compared to no sweetener. This finding aligns with prior research indicating that Chinese consumers express greater concern about sweetener safety compared to the Western consumers [42]. Finally, the estimated standard deviations for all random parameters reveal the presence of statistically significant heterogeneity in adolescent preferences across all attributes and levels.

As for the interaction effects between social media and beverage attributes, our results indicate that social media exposure has significant interactions with preferences related to price, beverage type, and sweetener type. Specifically, a significant positive interaction effect is noted between social media usage and price. This suggests that longer social media exposure is associated with lower price sensitivity to beverages among adolescents. Furthermore, a significant negative interaction is noted between social media usage and opt-out, indicating that longer social media exposure is associated with a stronger preference for choosing sugar-sweetened beverages over opting out among adolescents. As for the beverage types, a significant positive interaction is observed between social media usage and carbonated drinks. This implies that longer social media exposure is associated with a stronger preference for choosing carbonated drinks over tea drinks among adolescents. Moreover, adolescents with longer social media exposure tend to have a lower preference for fruit juice beverages and a higher preference for the two types of sweeteners.

### 4.2. Urban–Rural Differences in Social Media Exposure and Adolescents’ SSB Preferences

Results based on subsamples stratified by urban–rural status show that the association between social media exposure and adolescents’ SSB preferences is heterogeneous across urban and rural areas. For rural adolescents (Table 3, Column 2), a significantly negative association is observed between social media exposure and the preference for the opt-out option, indicating that prolonged exposure correlates with a greater inclination to choose sugar-sweetened beverages. For beverage categories, a significant positive association is observed between social media exposure and carbonated drinks, but a negative association is found with juice. For sweetener types, it is associated with an increased preference for beverages containing both natural caloric and non-caloric artificial sweeteners.

For urban adolescents (Table 3, Column 4), the analysis shows a positive association between social media exposure and price-related preferences, suggesting that longer social media exposure is associated with lower price sensitivity to beverages among adolescents. For beverage categories, a significantly positive association is observed solely with carbonated drinks. As for sweetener types, social media exposure is associated only with preference for natural sweeteners. These findings imply that interventions to restrict social media exposure could help reduce SSB preferences among rural adolescents and increase price sensitivity among their urban counterparts.

### 4.3. Hurdle Model Estimates for the Selection of SSBs

Table 4 presents the results from the hurdle model, examining the association between social media exposure and adolescents’ SSB choice. Results from the Probit model indicate that longer usage time of social media is associated with a higher likelihood of adolescents choosing any SSB over non-sweetened bottled water. Results from the Poisson model further demonstrate that increased time on social media is linked to a higher probability of SSB selection. For individual demographic characteristics, the amount of weekly allowance has a significant positive effect on adolescents’ SSB choice decisions, implying that greater income increases the likelihood of selecting SSBs. Family food environment is also significantly associated with adolescents’ SSB consumption [43]. Specifically, individuals are more inclined to choose SSBs when beverages are highly available at home. Interestingly, adolescents with a higher Body Mass Index (BMI) show a tendency to avoid choosing SSBs. Consistent with previous research, several factors are associated with a higher frequency of SSB choices; these factors include being male, having an older father, and belonging to a larger household [44].

### 4.4. The Impact of Social Media on Adolescents’ SSB Consumption

For adolescents’ actual consumption behavior, the OLS model is employed to analyze the association between social media and their SSB consumption. The results presented in Table 5 indicate that the coefficient for social media exposure in relation to adolescents’ SSB consumption is 0.188, significant at the 1% level. This indicates a significant positive association between social media exposure and adolescents’ SSB consumption, and longer social media exposure is associated with greater SSB consumption among adolescents. For individual characteristics, male adolescents report greater SSB consumption. Furthermore, both a larger allowance amount and higher availability of SSBs at home are associated with increased consumption among adolescents. We conduct robustness checks using a Tobit model and 1% winsorization, and the results support the original findings. In summary, we find that social media exposure is consistently associated with adolescents’ SSB choices and consumption behaviors. Therefore, we have to pay attention to the links between social media exposure and adolescents’ unhealthy consumption and take measures to protect the health interests of the adolescent population.

## 5. Discussion

This study explores the relationship between social media and two themes: adolescents’ SSB choice preferences and consumption. It applies mixed logit, hurdle models, and Ordinary Least Squares (OLS) models to survey data from adolescents in China. The results show that social media exposure is significantly associated with adolescents’ preferences for SSB product attributes, and that this association differs between urban and rural areas. Furthermore, social media exposure is significantly and positively associated with the quantity of SSB consumption among adolescents.

Consistent with prior research, the use of social networking is associated with a higher likelihood of unhealthy dietary behaviors among adolescents [45,46]. Social media marketing influences children’s dietary choices, promoting the request, purchase, selection, and intake of unhealthy foods and beverages [47]. Adolescents who are more sensitive to advertising not only show a stronger preference for SSBs, but this preference also more strongly impacts their actual consumption [48]. Kucharczuk et al. [28] find that adolescents often consume more unhealthy foods and beverages after watching food and beverage brand videos on social media platforms. The prolonged use of smartphones and the internet for accessing social media leads to increased SSB intake among adolescents [49]. Previous research on other types of food marketing demonstrates that exposure to media depicting unhealthy products is associated with increased consumption of such products [50].

This study reveals the association between social media exposure and adolescents’ preferences for SSB attributes and clarifies that restrictions on social media should be coordinated with different policies across urban and rural areas. Researchers are showing increasing attention to the topic of SSB taxation. Some argue that such taxation positively impacts the reduction of SSB consumption [51], while others contend that it affects adult beverage consumption but does not significantly impact children’s consumption [52]. From the perspective of consumption preferences, this study gives some more precise conclusions. We suggest that SSB taxation is associated with changes in beverage consumption preferences among adolescents in rural areas, whereas for adolescents in urban areas, a combination of SSB taxation and social media restrictions may be more effective. Additionally, longer social media usage is associated with a greater tendency for adolescents to choose SSBs, which ultimately leads to an increase in SSB intake. This study emphasizes the potential value of implementing measures to regulate adolescents’ social media usage.

This study has several limitations. Firstly, when exploring the association between social media exposure and adolescents’ preferences for SSB, it is not possible to exhaust all attributes of SSBs. The preference data from the discrete choice experiment are based on stated preferences rather than revealed preferences or actual behavior. Due to the hypothetical nature of the choice scenarios presented in the survey, preference estimates derived from the mixed logit model may not fully reflect actual sugar-sweetened beverage consumption behaviors. Second, due to data availability constraints, the measurement of SSB consumption is based only on consumption frequency and portion size, as we cannot obtain information on the sugar content of different SSBs. This study cannot make strong causal claims, as potential omitted variables may cause endogeneity. Third, the sample is drawn only from parts of Henan Province and is not nationally representative. Therefore, one must be cautious when generalizing the findings to other regions or the whole country. Future research should validate the results in broader geographic areas. Future research could combine the mixed logit framework with revealed preference data or real economic incentives to address the potential hypothetical bias of the stated choice method used in this study.

## 6. Implications and Conclusions

### 6.1. Implications

The findings of this study suggest practical implications for public health interventions aimed at reducing SSB consumption among adolescents. Given that increased social media use is associated with higher preferences and choice of SSBs, parents, educators, and policymakers should consider strategies to mitigate exposure to unhealthy beverage marketing online. Potential applications include the development of digital literacy programs that educate adolescents on the persuasive techniques used in social media advertising and the implementation of platform-level policies restricting targeted marketing of sugary drinks to minors. Additionally, parental monitoring of social media usage may serve as practical measures to reduce adolescents’ SSB intake. Overall, these strategies can help counteract the negative influence of social media on adolescent dietary behaviors and support a healthier lifestyle.

### 6.2. Future Research

Future research can examine causal associations more rigorously by using panel data, employing instrumental variable methods, or treating changes in social media policies as exogenous shocks. Studies can also test students’ actual behavioral responses to different sugar-labeling schemes or price subsidies/taxes in retail settings or school cafeterias and compare these findings against those from discrete choice experiments.

### 6.3. Conclusions

This study explores the association of product attributes, such as price, sugar content, beverage type, and sweetener type with beverage choice, as well as the role of social media use in adolescents’ selection of such beverages, thereby enriching the existing literature. Our results show that characteristics such as price, sugar content, and sweetener type are important in adolescents’ beverage choices, and adolescents with different levels of social media use exhibit different beverage preferences. Moreover, although the association between social media exposure and beverage product attributes differs between urban and rural areas, both contexts underscore the necessity of limiting adolescents’ social media use. Similarly, greater social media exposure is associated with both a higher likelihood and frequency of choosing SSBs among adolescents. This finding highlights the tangible benefits of social media restrictions.

## Figures and Tables

**Table 1:** Descriptive statistics.

Variable	Sample Size	Define	Min	Max	Mean	SD
Beverage	1517	Average weekly amount (mL)	0	16,500	798.385	1042.772
Webtime	1517	0 = None 1 = Less than 1 h 2 = 1–2 h 3 = 2–3 h 4 = 3–4 h 5 = 4 h or more	0	5	3.537	1.536
Gender	1517	Female = 0; Male = 1	0	1	0.511	0.500
Age	1517	Years	12	16	13.63	0.776
Ethnicity	1517	1 = Han; 0 = Ethnic Minorities	0	1	0.983	0.130
Rural	1517	rural = 1; urban = 0	0	1	0.747	0.435
Lodge	1517	non-boarders = 0; Boarders = 1	0	1	0.221	0.415
Household	1517	Permanent residents	2	12	5.386	1.288
Fa age	1517	Years	30	61	40.38	5.019
Mo age	1517	Years	30	61	39.14	4.894
Alledu	1517	No formal schooling = 1, Primary school = 2, Junior secondary school = 3, Senior secondary school = 4, University = 5, Postgraduate = 6	1	6	3.606	0.788
Pocketmoney	1517	amount of monthly allowance	0	500	32.817	39.845
BMI	1517	kg/m^2^	12.55	35.66	19.22	2.890
Available	1517	1 = Never, 2 = Rarely, 3 = Sometimes, 4 = Often, 5 = Always	1	5	2.461	0.905

Webtime = Daily social media usage; Fa age = Father’s age; Mo age = Mother’s age; Alledu = Highest level of parents’ education; BMI = Body Mass Index.

**Table 2:** Mixed logit regression results for discrete choice experiments.

	(1)	(2)
	Main Effect	Interaction
Mean		
price	−0.274 ***	0.017 **
	(0.026)	(0.007)
out	−1.230 ***	−0.106 **
	(0.184)	(0.047)
sweetne	−0.037 ***	0.003
	(0.012)	(0.003)
CocolBever	−0.879 ***	0.173 ***
	(0.183)	(0.046)
JuiceBever	0.294 *	−0.069 *
	(0.150)	(0.038)
EnergyBever	−0.773 ***	−0.033
	(0.190)	(0.048)
Nature	−0.603 ***	0.067 **
	(0.110)	(0.028)
Artif	−0.809 ***	0.151 ***
	(0.125)	(0.032)
SD		
sweetne	0.104 ***	
	(0.004)	
CocolBever	1.907 ***	
	(0.086)	
JuiceBever	1.388 ***	
	(0.072)	
EnergyBever	1.924 ***	
	(0.084)	
Nature	0.408 ***	
	(0.087)	
Artif	0.449 ***	
	(0.090)	
Number of obs.	36,408	
Log-likelihood	−11,234	
AIC	22,513	
BIC	22,700	

AIC = Akaike information criterion; BIC = Bayesian information criterion; Nature = natural caloric sweeteners; Artif = non-caloric artificial sweeteners. Standard errors in parentheses * *p* < 0.1, ** *p* < 0.05, *** *p* < 0.01.

**Table 3:** Regression results based on urban–rural differences.

	Rural		Urban	
	(1)	(2)	(3)	(4)
	Main Effect	Interaction	Main Effect	Interaction
Mean				
price	−0.255 ***	0.006	−0.327 ***	0.047 ***
	(0.031)	(0.008)	(0.051)	(0.013)
out	−1.039 ***	−0.178 ***	−1.738 ***	0.094
	(0.215)	(0.055)	(0.353)	(0.091)
sweetne	−0.033 **	0.002	−0.046 **	0.005
	(0.015)	(0.004)	(0.023)	(0.006)
CocolBever	−0.745 ***	0.128 **	−1.332 ***	0.321 ***
	(0.213)	(0.053)	(0.360)	(0.090)
JuiceBever	0.420 **	−0.100 **	−0.065	0.007
	(0.176)	(0.045)	(0.289)	(0.075)
EnergyBever	−0.643 ***	−0.069	−1.016 ***	0.046
	(0.225)	(0.057)	(0.361)	(0.091)
Nature	−0.598 ***	0.066 **	−0.617 ***	0.069
	(0.129)	(0.032)	(0.212)	(0.054)
Artif	−0.789 ***	0.145 ***	−0.807 ***	0.159 ***
	(0.145)	(0.037)	(0.240)	(0.061)
SD				
sweetne	0.107 ***		0.095 ***	
	(0.005)		(0.009)	
CocolBever	1.893 ***		1.915 ***	
	(0.101)		(0.172)	
JuiceBever	1.400 ***		1.400 ***	
	(0.083)		(0.147)	
EnergyBever	1.948 ***		1.835 ***	
	(0.099)		(0.162)	
Nature	−0.390 ***		0.499 ***	
	(0.103)		(0.147)	
Artif	0.409 ***		0.412 **	
	(0.119)		(0.199)	
Number of obs.	27,192		9216	
Log-likelihood	−8366		−2859	
AIC	16,775		5762	
BIC	16,956		5919	

Standard errors in parentheses * *p* < 0.1, ** *p* < 0.05, *** *p* < 0.01.

**Table 4:** The results of the hurdle model.

	(1)		(2)	
	Probit		Poisson	
	Coefficient	Std. Error	Coefficient	Std. Error
main				
webtime	0.066 ***	0.023	0.032 ***	0.005
gender	−0.015	0.070	0.022 *	0.012
age	−0.061	0.044	0.006	0.008
rural	−0.039	0.082	0.006	0.014
lodge	0.001	0.085	0.002	0.015
household	−0.040	0.028	0.008 *	0.005
fa age	0.021	0.016	0.005 *	0.003
mo age	−0.007	0.016	−0.002	0.003
alledu	−0.023	0.046	−0.005	0.008
pocketmoney	0.104 ***	0.026	−0.002	0.005
BMI	−0.019 *	0.011	−0.002	0.002
available	0.285 ***	0.055	0.074 ***	0.007
_cons	1.252 *	0.726	1.030 ***	0.126
Number of obs.	3034		2791	
Log-likelihood	−785.3		−5328.3	

Standard errors in parentheses * *p* < 0.1, ** *p* < 0.05, *** *p* < 0.01.

**Table 5:** Adolescents’ SSB consumption outcomes.

	(1)	(2)	(3)
	OLS	1% Winsorization	Tobit
Webtime	0.188 ***	0.188 ***	0.215 ***
	(0.042)	(0.042)	(0.050)
Gender	0.709 ***	0.703 ***	0.780 ***
	(0.120)	(0.120)	(0.141)
Age	0.075	0.075	0.090
	(0.075)	(0.075)	(0.089)
Ethnicity	−0.204	−0.212	−0.245
	(0.457)	(0.456)	(0.522)
Rural	0.073	0.076	0.092
	(0.136)	(0.136)	(0.161)
Lodge	−0.163	−0.160	−0.188
	(0.148)	(0.148)	(0.174)
Household	0.024	0.022	0.030
	(0.044)	(0.044)	(0.052)
Fa age	0.022	0.023	0.027
	(0.026)	(0.026)	(0.031)
Mo age	−0.018	−0.020	−0.022
	(0.025)	(0.025)	(0.029)
Alledu	−0.005	−0.004	−0.008
	(0.078)	(0.078)	(0.093)
Pocketmoney	0.008 ***	0.007 ***	0.008 ***
	(0.001)	(0.001)	(0.002)
BMI	−0.029	−0.029	−0.036
	(0.021)	(0.021)	(0.025)
Available	0.770 ***	0.763 ***	0.871 ***
	(0.064)	(0.064)	(0.077)
_cons	1.741	1.793	1.084
	(1.323)	(1.321)	(1.555)
var(e.beveragrml)			6.932 ***
			(0.440)
Number of obs.	1517	1517	1517
Log-likelihood	−3382	−3382	−3370

Standard errors in parentheses * *p* < 0.1, ** *p* < 0.05, *** *p* < 0.01.

## Data Availability

The data that support the findings of this study are available from the corresponding author upon reasonable request.

## Abbreviations

The following abbreviations are used in this manuscript:

SSB	Sugar-sweetened beverage
OLS	Ordinary Least Squares
DCE	Discrete choice experiment
BMI	Body Mass Index
